# Enrichment of Type I Methanotrophs with *nirS* Genes of Three Emergent Macrophytes in a Eutrophic Wetland in China

**DOI:** 10.1264/jsme2.ME19098

**Published:** 2020-01-23

**Authors:** Ju-mei Liu, Zhi-hua Bao, Wei-wei Cao, Jing-jing Han, Jun Zhao, Zhen-zhong Kang, Li-xin Wang, Ji Zhao

**Affiliations:** 1 College of Life Sciences, Inner Mongolia University, Hohhot, 010021, China; 2 Ministry of Education Key Laboratory of Ecology and Resource Use of the Mongolian Plateau & Inner Mongolia Key Laboratory of Grassland Ecology, School of Ecology and Environment, Inner Mongolia University, Hohhot, 010021, China; 3 Inner Mongolia Key Laboratory of Environmental Pollution Control & Waste Resource Reuse, Inner Mongolia University, Hohhot, 010021, China; 4 College of Chemistry and Environmental Engineering, Chongqing Key Laboratory of Environmental Materials & Remediation Technologies, Chongqing University of Arts and Sciences, Chongqing, 402160, China

**Keywords:** emergent macrophytes, root-associated Type I methanotrophs, denitrifiers, eutrophic wetland, CARD-FISH

## Abstract

The *pmoA* gene, encoding particulate methane monooxygenase in methanotrophs, and *nirS* and *nirK* genes, encoding bacterial nitrite reductases, were examined in the root and rhizosphere sediment of three common emergent macrophytes (*Phragmites australis*, *Typha angustifolia*, and *Scirpus triqueter*) and unvegetated sediment from eutrophic Wuliangsuhai Lake in China. Sequencing analyses indicated that 334 out of 351 cloned *pmoA* sequences were phylogenetically the most closely related to type I methanotrophs (*Gammaproteobacteria*), and *Methylomonas denitrificans*-like organisms accounted for 44.4% of the total community. In addition, 244 out of 250 cloned *nirS* gene sequences belonged to type I methanotrophs, and 31.2% of *nirS* genes were the most closely related to paddy rice soil clone SP-2-12 in *Methylomonas* of the total community. Three genera of type I methanotrophs, *Methylomonas*, *Methylobacter*, and *Methylovulum*, were common in both *pmoA* and *nirS* clone libraries in each sample. A quantitative PCR (qPCR) analysis demonstrated that the copy numbers of the *nirS* and *nirK* genes were significantly higher in rhizosphere sediments than in unvegetated sediments in *P. australis* and *T. angustifolia* plants. In the same sample, the *nirS* gene copy number was significantly higher than that of *nirK*. Furthermore, type I methanotrophs were localized in the root tissues according to catalyzed reporter deposition-fluorescence *in situ* hybridization (CARD-FISH). Thus, *nirS*-carrying type I methanotrophs were enriched in macrophyte root and rhizosphere sediment and are expected to play important roles in carbon/nitrogen cycles in a eutrophic wetland.

Eutrophication is a serious issue in major aquatic ecosystems worldwide. The control of increasing eutrophication and improvements in water quality continue to be a major global challenge (Ansari *et al.*, 2011). Aquatic macrophytes are known to play important roles in improving water quality (Dhote and Dixit, 2009) by removing considerable amounts of nitrogen and phosphorus from eutrophic bodies of water (Zheng *et al.*, 2018). In addition, the rate of methane (CH_4_) emission was found to be higher from strongly eutrophic ponds than from weakly eutrophic ponds (Merbach *et al.*, 1996), and this was attributed to anoxia in eutrophic lakes favoring the production of CH_4_ (Liikanen and Martikainen, 2003). *Phragmites* spp., *Typha* spp., and *Scirpus* spp. are three common emergent types of macrophyte vegetation that are distributed worldwide (Vymazal, 2013), and mediate CH_4_ emissions from wetlands to the atmosphere (Grünfeld and Brix, 1999). The root zone (root and rhizosphere sediment) of wetland plants is under aerobic conditions (Armstrong *et al.*, 2000) and allows the growth of aerobic methanotrophs (Type I and Type II) that utilize methane and methanol as their sole carbon and energy sources (Hanson and Hanson, 1996). Previous studies suggested that methylotrophic bacteria and other bacterial groups live in the root zones of *Phragmites australis* and *Typha latifolia* (Chen *et al.*, 2012; Fausser *et al.*, 2012); however, it currently remains unclear whether differences exist in the community structure of root-associated methanotrophs among plant species, and how these methanotrophs are distributed in the root tissues of macrophytes has not yet been clarified.

Aerobic methanotrophs form an important bridge between the global carbon and nitrogen cycles, a relationship that is impacted by nitrogen deposition and deficiency (Bodelier and Laanbroek, 2004; Stein and Klotz, 2011; Bao *et al.*, 2014b; Ikeda *et al.*, 2014). The findings of metaproteomic analyses suggest that methane oxidation and nitrogen fixation under low-nitrogen conditions occur inside and on the surface of rice roots via type II methanotrophs, including *Methylosinus* spp. (Bao *et al.*, 2014a; Minamisawa *et al.*, 2016). However, type I methanotrophs have been the main group detected on the surface of aquatic plants (Yoshida *et al.*, 2014), and they predominate in environments with high nitrogen loads (Beck *et al.*, 2013). In recent years, the role of aerobic methanotrophs in mediating the nitrogen cycle, either indirectly or directly, has been attracting increasing attention. Modin *et al.* (2010) reported the genetic potential for denitrification by type I methanotrophs in mixed culture biofilms in membrane biofilm reactors. Stein and Klotz, (2011) inferred a genome inventory for the denitrifying pathway in methanotrophic isolates. Furthermore, the denitrification activity of individual type I methanotrophic isolates was reported by Kits *et al.* (2015a; 2015b). Thus, aerobic methanotrophs employ two processes for denitrification: 1) two strains (*i.e.* *Methylomonas denitrificans* strain FJG1 and *Methylomicrobium album* strain BG8) perform direct denitrification under anoxic conditions in single cells (Kits *et al.*, 2015a; 2015b); and 2) aerobic methanotrophs and denitrifiers, which form consortia, also utilize methane as an external carbon source for denitrification via the aerobic methane oxidation coupled to denitrification (AME-D) process (Zhu *et al.*, 2016). In both cases, aerobic methanotrophs appear to provide electron donors to the denitrification reaction. Although type I methanotrophs have been detected as the dominant group on aquatic plant surfaces, the genetic potential for the denitrification of methanotrophs in natural wetlands/ecosystems has not yet been examined in detail.

Denitrification is a beneficial process in the eutrophication aquatic system. Conventional primers for *nirS* and *nirK* genes (encoding nitrite reductase enzymes) that have some limitations in detecting diverse *nirS*/*nirK*-type environmental denitrifiers have been reported. The *Methylomonas*, *Methylobacter*, *Methylosarcina*, *Methylomicrobium*, and *Methylovulum* genera of Type I methanotrophs possess functional denitrification genes, including *nirS* or *nirK* (Stein and Klotz, 2011; Padilla *et al.*, 2017). Methanotrophic denitrifiers from paddy soil have been detected using primers, including the primer set nirSC2F/nirSC2R (Wei *et al.*, 2015). These primers are expected to promote the detection of methanotrophic denitrifiers in wetland ecosystems, including macrophytes.

Wuliangsuhai (WLSH) Lake is located near the city of Bayannur in the Inner Mongolia Autonomous Region in China. This lake is the largest freshwater lake in the Yellow River watershed; it is a rare, large-scale, shallow lake and grass wetland in this arid region. It has important ecological functions that maintain water resources, regulate the floods and droughts associated with an arid climate, and provide high biological diversity as a Ramsar site (Borruso *et al.*, 2017). The lake recently became eutrophic after receiving large volumes of irrigation drainage water as well as municipal and industrial wastewater with high nitrogen and phosphorus contents from the Hetao Basin (Wu *et al.*, 2017). *Phragmites australis* (common reed), *Typha angustifolia* (narrow leaf cattail), and *Scirpus triqueter* (bulrush) are the dominant macrophytes of WLSH (Duan *et al.*, 2005). Many studies have examined microorganisms in their rhizosphere sediments, including the methylotroph- and heterotroph-mediated processes of carbon and other element cycles (Borruso *et al.*, 2017). However, few studies have focused on the characteristics of methanotrophic communities in the roots of macrophytes or on denitrification by aerobic methanotrophs themselves in natural wetlands.

In the present study, we (i) examined the abundance and diversity of methanotrophs and denitrifying bacteria in the root zones of three emergent macrophytes, *P. australis*, *T. angustifolia*, and *S. triqueter*, as well as plant-free sediment from WLSH, a eutrophic wetland; (ii) identified which methanotroph groups had genetic potential for denitrification; and (iii) clarified the localization of methanotrophs in the root tissues of these three macrophytes.

## Materials and Methods

### Macrophyte roots, sediment, and water sampling and an analysis of physicochemical properties

**Fig. 1.**
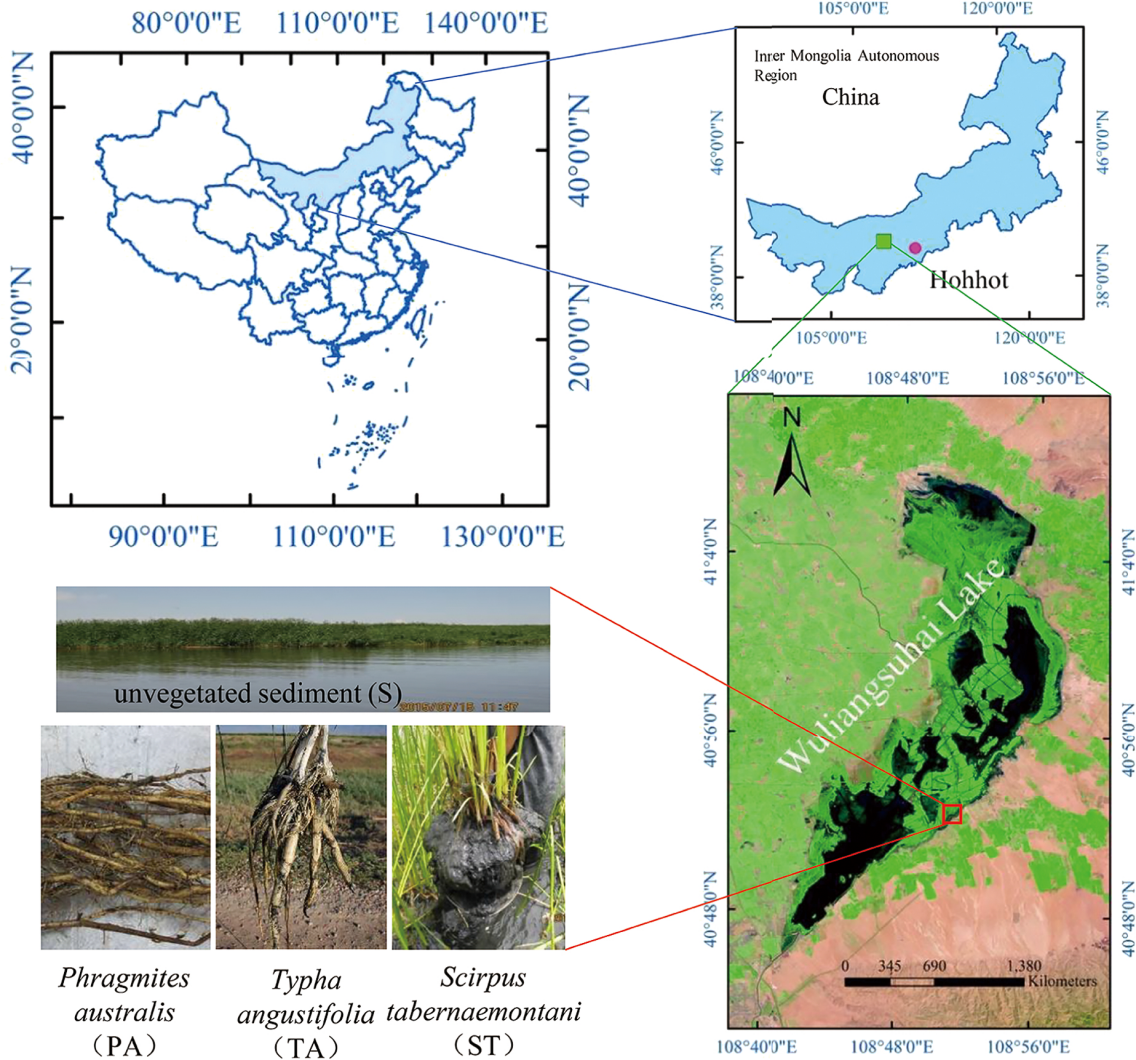
Sampling site diagram of three macrophytes and unvegetated sediment from the Wuliangsuhai wetland.

Three plants each of *P. australis* (PA), *T. angustifolia* (TA), and *S. triqueter* (ST) were collected along with soil blocks from the littoral wetland of WLSH Lake (latitude 40°47′–41°03′N, longitude 108°43′–108°57′E) during the growing season on July 15^th^, 2015 (Fig. 1). Plant roots and rhizosphere sediments were sampled according to a previous study (Kimura, 2004). Briefly, after the plants with soil blocks were sampled, soil blocks including plants were divided vertically into two equal parts to collect the roots. Some of the exposed roots were carefully picked from the plants using sterilized forceps and placed into a 50-mL centrifuge tube containing sterile water. Rhizosphere sediment samples were obtained before and after sonication (5–10 min). After the rhizosphere sediment was removed, the root samples were transferred to new centrifuge tubes (50 mL) containing sterile water and centrifuged at 8,000×*g* at 4°C for 10 min. All samples were stored at –‍80°C for later molecular analyses.

Unvegetated sediment (S) was sampled in triplicate from depths of 10–20 cm along with water from non-vegetated areas (Fig. 1). Sediment cores were packed in sterile plastic bags and transported immediately to the laboratory in an ice-cooled box. Sediment samples were air-dried at room temperature and passed through a 2-mm sieve for the analysis of physicochemical properties, including pH, electrical conductivity (EC), total organic carbon (TOC), total nitrogen (TN), total phosphorus (TP), nitrate nitrogen (NO_3_^–^-N), and ammonium nitrogen (NH_4_^+^-N); the same properties were measured in water samples. Soil pH was assessed using a pH meter (HQ40D; Hach) in a 1:2.5 (w/v) suspension in ultrapure water. EC was measured in a soil-water suspension (1:2.5 [w/v]) using a conductivity meter (Leici DDS-307), and this was used to evaluate salinity (Wollenhaupt *et al.*, 1986). TOC was assessed by dichromate oxidation. TN was measured using the Kjeldahl method. TP was measured using the ammonium molybdate spectrophotometric method. NO_3_^–^-N content was assessed by UV spectrophotometry, and NH_4_^+^-N content was measured by indophenol blue colorimetry (Nelson and Sommers, 1982; Bao, 2000).

### Nucleic acid extraction

Genomic DNA was extracted from 0.5–0.8 g of sediment or root samples using the Fast DNA SPIN Kit for Soil (MP Biomedicals) according to the manufacturer’s protocol. Regarding root samples, frozen tissues were ground into a powder using a mortar and pestle under liquid nitrogen before DNA extraction. DNA concentrations were quantified using a NanoPhotometer P-Class P-330C instrument (IMPLEN). Extracted DNA was immediately stored at –‍20°C.

### Clone library construction and phylogenetic analysis

Clone libraries were constructed for roots and sediments using the primer sets A189F/mb661R for the *pmoA* gene (Costello and Lidstrom, 1999) and nirSC2F/nirSC2R for the *nirS* gene (Wei *et al.*, 2015) (Table S3). PCR amplification was performed with 50-μL mixtures in 0.2-mL tubes using a Mastercycler (Eppendorf). The reaction mixtures included 5.0 μL 10× PCR buffer (plus Mg^2+^), 4.0 μL 250‍ ‍mM each dNTP, 0.4 μL 5 U μL^–1^ *Ex Taq* DNA polymerase (Takara Biotech) plus 1.0 μL containing 10‍ ‍mM of each primer and 1.0 μL bovine serum albumin (BSA; Amesco; 20‍ ‍mg‍ ‍mL^–1^). BSA was added to reduce inhibition by humic substances (Kreader, 1996). Amplification was performed with an initial denaturation step at 95°C for 3 min followed by 35 cycles at 95°C for 1 min, 55°C for 1 min, and 72°C for 40‍ ‍s (for *pmoA*), or at 95°C for 10 min, followed by 35 cycles at 95°C for 30‍ ‍s, 56°C for 30‍ ‍s, and 72°C for 30‍ ‍s (for *nirS*), with a final extension at 72°C for 10 min for both procedures. Regarding each plant species, three PCR products were mixed at equal molar ratios, and these formed a composite PCR product. Composite PCR products were then purified using a Gene JET PCR Purification Kit (Promega) and ligated into the pGEM-T Easy Vector (Promega) according to the manufacturer’s instructions. All clones were sequenced by the Sanger method. Nucleic acid sequences were translated with MEGA software, version 5.2.2 (Tamura *et al.*, 2011). After alignment, amino acid sequences were clustered into operational taxonomic units (OTUs) at ≥91% amino acid identity for the *pmoA* gene (Heyer *et al.*, 2002) or ≥90% amino acid identity for the *nirS* gene using mothur software (Schloss *et al.*, 2009). Diversity indices (*e.g.* coverage, Chao1, ACE, Shannon, and Simpson indices) were estimated by mothur. Representative sequences for each OTU were selected by mothur software by default, and these sequences were the most abundant within each OTU. Phylogenetic analyses of the representative sequences for each OTU were performed using MEGA with the neighbor-joining method. Bootstrap values were based on 1,000 replications.

### Quantification of *pmoA*, *nirS*, and *nirK* genes

qPCR assays were performed in 96-well polypropylene plates on a CFX Connect Optical Real-Time Detection System (Bio-Rad Laboratories). Regarding each plant species, DNA samples from three plants, serving as three biological replicates, were used for qPCR of the target genes. Three technical replicates were run for each DNA sample. qPCR reactions were performed in 20-μL reaction mixtures containing 10 μL 2× SYBR Premix Ex Taq (Takara Biotech), 500 nM of each primer, and 10–20 ng DNA templates. Blanks included sterile ultrapure water as the template instead of extracted sample DNA. Details on the primers used and reaction conditions for qPCR assays are provided in Table S3. We used the methanotroph-specific primer set A189F/mb661R for the *pmoA* gene (Costello and Lidstrom, 1999), the denitrifying bacteria primer set nirSC2F/nirSC2R for the *nirS* gene (Wei *et al.*, 2015), and the primer set F1aCu/R3Cu for the *nirK* gene (Hallin and Lindgren, 1999). All primers were purchased from Sangon Biotech.

Standard curves for qPCR assays were generated as described previously (Cai *et al.*, 2016) and constructed using a 10-fold dilution series of plasmids harboring the target gene. Plasmids were extracted using the TIANprep Mini Plasmid Kit (Tiangen Biotech); concentrations were measured using a NanoPhotometer P-Class P-330C (IMPLEN) and used to calculate standard copy numbers. Amplification efficiency ranged between 93.1 and 97.0% for *pmoA*, 94.0 and 96.3% for *nirS*, and 90.2 and 93.2% *nirK*. R^2^ values for standard curves ranged between 0.996 and 1.000 for the three genes. A melting curve analysis was used to confirm the specific amplification of target genes and always showed a single peak.

### CARD-FISH

Catalyzed reporter deposition-fluorescence *in situ* hybridization (CARD-FISH) is a powerful tool in modern microbial ecology and has strong signal sensitivity (Kubota, 2013). It has been used to directly detect methanotrophs in paddy soil and field-grown rice roots (Bao *et al.*, 2014a; Cai *et al.*, 2016). *M. koyamae* strain Fw12E-Y^T^ (NCIMB14606) was used as a positive control. This strain was incubated in 1a medium at 30°C with 50% (v/v) methane in the headspace (Ogiso *et al.*, 2012). *Thiobacillus thiooxidans* (JCM 3867) was used as a negative control (Eller *et al.*, 2001).

To detect type I methanotrophs in the root tissues of PA, TA, and ST, we used probes for Mγ84 (3′-AGCCCGCGACTGCTCACC-5′) and Mγ705 (3′-CTAGACTTCCTTGTGGTC-5′) (Eller *et al.*, 2001). These probes were labeled with horseradish peroxidase at the 5′ end (Japan Bio Services). Plant roots were sectioned into 2-‍cm-thick pieces to visualize the location of type I methanotrophs. CARD-FISH analyses were performed as previously described (Bao *et al.*, 2014a), with minor modifications. Briefly, endogenous peroxidases were inactivated with 1.2% H_2_O_2_ in methanol at room temperature (RT) for 60 min instead of 0.15% H_2_O_2_ in methanol for 30 min as described previously.

We used an epifluorescence microscope (Ci-L; Nikon) for microscopic observations and image acquisition. To visualize *M. koyamae* strain Fw12E-Y^T^, we counterstained samples with 4′,6-diamidino-2-phenylindole (DAPI; 1 μg mL^–1^) at RT for 2 min. We viewed Mγ84+Mγ705-positive cells in the roots using a laser scanning confocal microscope (LSM 710; Carl Zeiss) and ZEN 2012 software (Carl Zeiss).

### Statistical analysis

We tested for significant differences in *pmoA*, *nirS*, and *nirK* gene numbers by a one-way ANOVA with the least significant difference (LSD) test. We used Pearson’s linear correlation to assess whether a correlation exists between *pmoA* and *nirS* gene copies. All statistical analyses were conducted using SPSS software, version 19.0 (IBM), and the significance level was *P*<0.05. We applied the chi-squared test to examine the significance of differences in the proportion of OTU or bacteria taxa between PA plants and TA or ST plants. We used Canoco software, version 4.5 (Ithaca) for the principal component analysis (PCA).

### Nucleotide sequence accession numbers

All cloned sequences were deposited in the GenBank (http://www.ncbi.nlm.nih.gov/BankIt/) nucleotide sequence database under accession numbers MG016967–MG017151 (roots), MG017152–MG017207 (unvegetated sediment), and MG017208–MG017317 (rhizosphere sediments) for the *pmoA* gene, and MG016713–MG016823 (roots), MG016724–MG016726, MG016728–MG016752, MG016754–MG016757, and MG016760 (unvegetated sediment), and MG016861–MG016966 (rhizosphere sediments) for the *nirS* gene.

## Results

### Physicochemical properties of WLSH Lake

Samples were collected from WLSH Lake in the Inner Mongolia Autonomous Region of China (Fig. 1). The properties of water and sediment from WLSH Lake are shown in Table S1. The lake had saline-alkaline conditions, with the pH and EC of the water and sediment ranging between 9.0 and 9.3 and 1.35 and 6.25 ds m^–1^, respectively. The high concentrations of TOC (up to 27.62 mg L^–1^ in water and 18.35 g kg^–1^ dry weight in sediment), TP (up to 0.03 mg L^–1^ in water and 0.62 g kg^–1^ in sediment), TN (up to 1.87 mg L^–1^ in water and 2.60 g kg^–1^ in sediment), and nitrogenous compounds, particularly ammonium (up to 0.30 mg L^–1^ in water and 24.45 mg kg^–1^ dry weight in sediment) and nitrate (up to 0.19 mg L^–1^ in water and 1.28 mg kg^–1^ dry weight in sediment), in both the water and sediment samples showed that the lake was experiencing severe eutrophication.

### Phylogenetic diversities of methanotrophs based on the *pmoA* gene

**Fig. 2.**
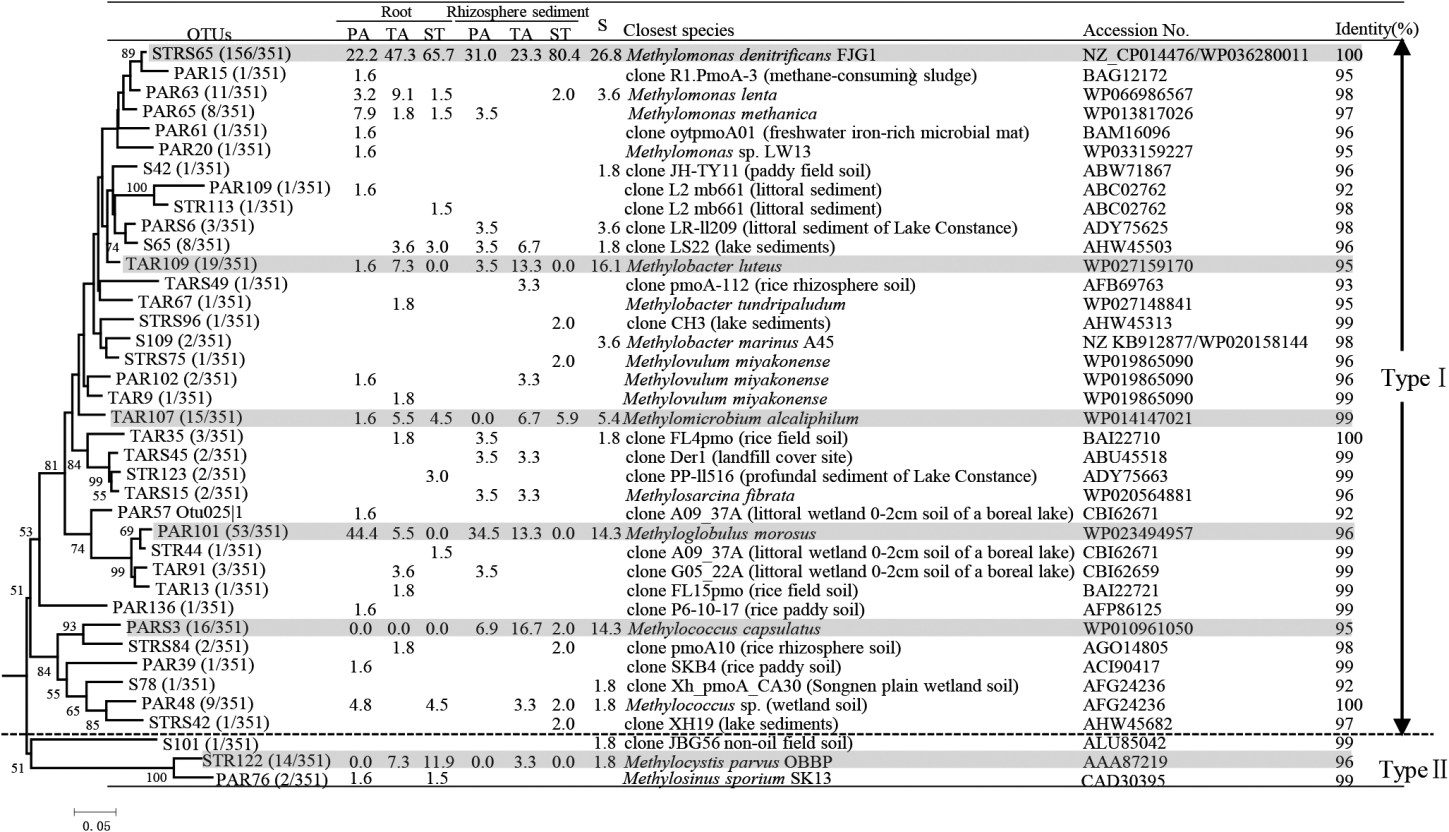
Phylogenetic tree of representative OTUs (91% amino acid identity) based on translated *pmoA* gene sequences from roots and rhizosphere sediments of three plants and unvegetated sediment (PA, *Phragmites australis*; TA, *Typha angustifolia*; ST, *Scirpus triqueter*; R, root; RS, rhizosphere sediment; S, unvegetated sediment). The table lists the relative abundance of clones belonging to each OTU in each library and the results of BLAST searches using the representative sequences. The numbers in parentheses for each OTU indicate the number of clones out of the total number of clones. Gray shading indicates the main OTUs for roots and sediments.

**Fig. 3.**
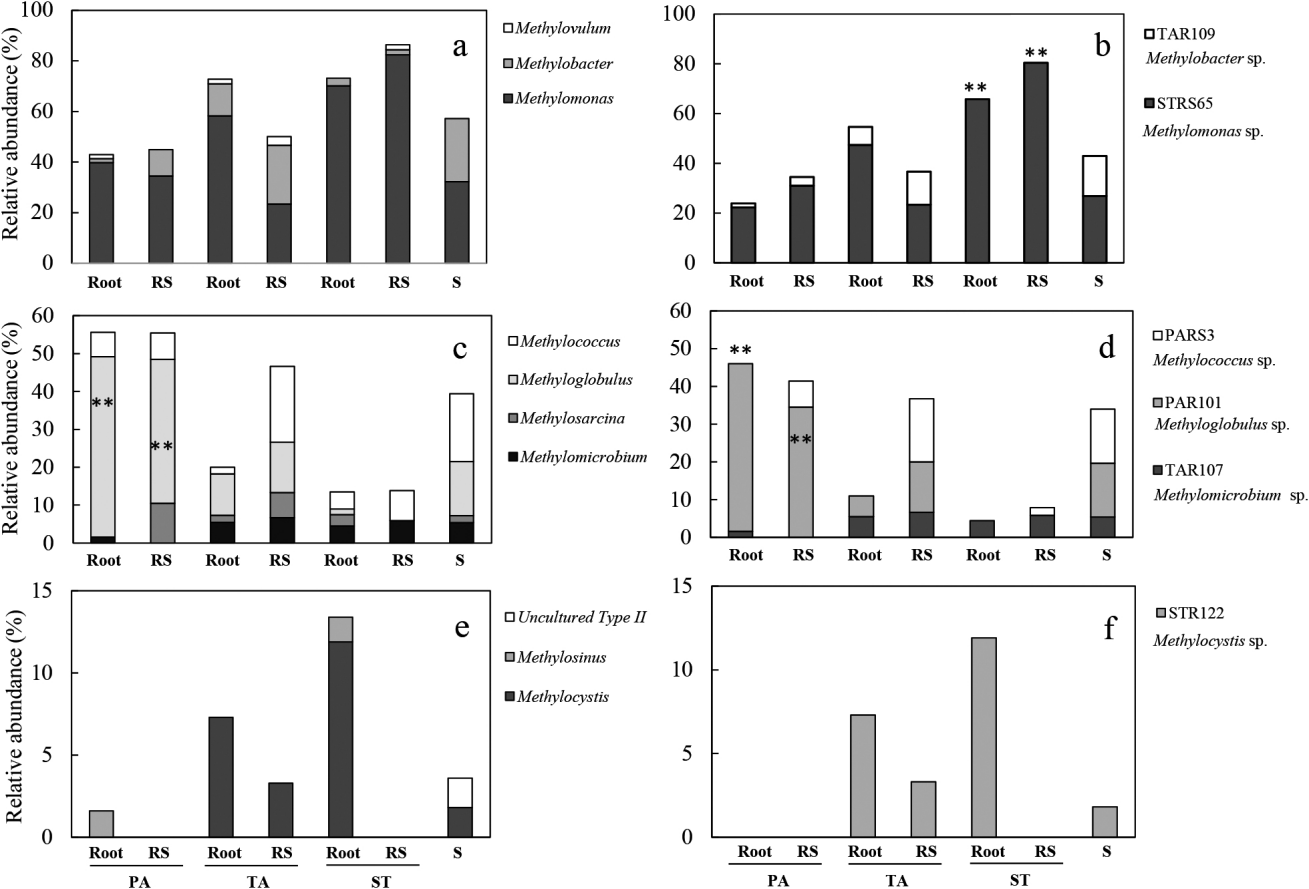
Phylogenetic compositions of *pmoA* gene clone libraries from roots and rhizosphere sediments of three plants and unvegetated sediments (PA, *Phragmites australis*; TA, *Typha angustifolia*; ST, *Scirpus triqueter*; RS, rhizosphere sediment; S, unvegetated sediment). (a, c, and e) Phylogenetic compositions at the genus level. (b, d, and f) Relative abundance of the main operational taxonomic units (OTUs) that occurred in the *pmoA* gene clone libraries. a and b: *Methylomonas*, *Methylobacter* and *Methylovulum* within type I methanotrophs, which were also frequently detected in the *nirS* gene clone libraries (Fig. 4); c and d: relative abundance of other type I methanotrophs, which were absent in the *nirS* gene clone libraries (Fig. 4); e and f: Type II methanotrophs, which were absent in the *nirS* gene clone libraries (Fig. 4). The abundance of each OTU (defined by ≥91% identity) corresponds to the data in Fig. 2. ** indicates a significant difference between PA plants and AT or ST plants at *P*<0.05, calculated with the chi-squared test.

Three hundred and fifty-one *pmoA* sequences were used to identify aerobic methanotroph diversity in the root zones of the PA, TA, and ST plants as well as plant-free sediment. Basic information regarding the *pmoA* clone library is shown in Table S2. A phylogenetic analysis of all sequences revealed that the majority of sequences belonged to the type I methanotrophs of seven genera: *Methylomonas*, *Methylobacter*, *Methylovulum*, *Methylomicrobium*, *Methylosarcina*, *Methyloglobulus*, and *Methylococcus* (334 sequences, 95.2%), with type II methanotrophs accounting for a minority (17 sequences, 4.8%) (Fig. 2). Further analyses at finer levels showed that the relative abundance of *Methylomonas* among all samples ranged between 23.3 and 82.4%. *Methylomonas* was clearly dominant over *Methylobacter* (1.6–23.3%) and *Methylovulum* (0–3.3%) (Fig. 3a). The relative abundance of *Methylococcus* in roots (1.8–6.4%) was lower than that in all sediments (including rhizosphere sediments and unvegetated sediment) (6.9–20.0%). Notably, the abundance of *Methyloglobulus* was higher in the root zone of PA (38.0–47.6%) than in those of TA (10.9–13.3%) and ST (0–1.5%) (Fig. 3c). In contrast, *Methylocystis* was mainly responsible for the abundance of type II methanotrophs in the root zones of both TA (3.3–7.9%) and ST (0–11.9%), but was not detected in the root zones of PA (0%) (Fig. 3e).

The phylogenetic analysis revealed that the relative abundance of OTU STRS65 was higher in ST than in PA, TA, and unvegetated sediment. In contrast, OTU TAR109 was only absent in ST (Fig. 3b). These two OTUs were similar to *M. denitrificans* strain FJG1 (100% sequence identity) (WP036280011) and *Methylobacter luteus* (95% sequence identity) (WP027159170), respectively (Fig. 2). The relative abundance of OTU TAR107 among all samples ranged between 1.6 and 6.7%; however, it was absent in the rhizosphere sediment of PA. The relative abundance of OTU PAR101 was higher in PA (34.5–44.4%) than in TA and unvegetated sediment (5.5–14.3%). Moreover, the relative abundance of OTU PARS3 was higher in the four sediment samples (Fig. 3d). The representative sequences of these three OTUs showed higher levels of identity than those of *M. alcaliphilum* (99%) (WP01414702), *Methyloglobulus morosus* (96%) (WP023494957), and *Methylococcus capsulatus* (95%) (WP010961050) (Fig. 2). The representative sequence of OTU STR122 was closely related to *Methylocystis parvus* strain OBBP (AAA87219), with 96% identity; this sequence was detected in TA and ST, but not in PA (Fig. 2 and 3f).

### Relationship of phylogenetic diversities between methanotrophs and denitrifiers based on *pmoA* and *nirS* genes

**Fig. 4.**
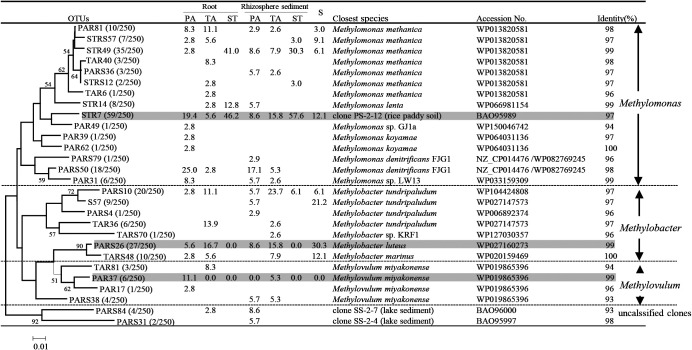
Phylogenetic tree of representative OTUs (90% amino acid identity) based on translated *nirS* gene clone sequences from roots and rhizosphere sediments of three plants and the unvegetated sediments. The table lists the relative abundance of clones belonging to each OTU in each library and the results of a BLAST search using representative sequences. The numbers in parentheses for each OTU indicate the number of clones out of the total clones. Gray shading indicates the main OTUs for roots and sediments.

**Fig. 5.**
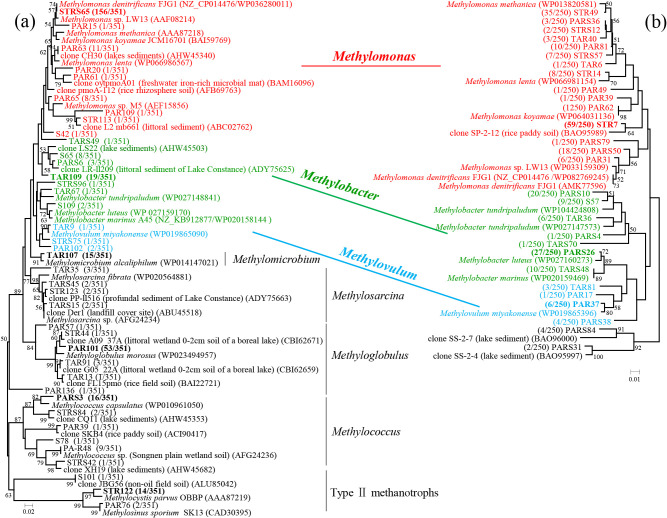
Phylogenetic relationships between *pmoA* and *nirS* gene clones. Phylogenetic trees were constructed based on translated (a) *pmoA* (91% amino acid identity) and (b) *nirS* (90%) gene clone sequences from the roots and rhizosphere sediments of three plants and unvegetated sediment using the neighbor-joining method. The numbers in parentheses for each OTU indicate the number of clones out of the total clones. The accession numbers of the reference strains in the GenBank database are indicated in brackets. The bootstrap values (≥50%) from 1,000 replicates are indicated next to the branches. *Methylomonas* (red), *Methylobacter* (green), and *Methylovulum* (blue) were commonly detected in both the *pmoA* and *nirS* gene clone libraries from all samples. Bold typeface indicates the main OTUs.

Two hundred and fifty *nirS* sequences were used to identify denitrifier diversity in the root zones of PA, TA, and ST plants as well as plant-free sediment. Basic information on the *nirS* clone library is also shown in Table S2. The phylogenetic analysis revealed that three OTUs (STR7, PARS26, and PAR37) were abundant in all samples in the *nirS* gene clone library (Fig. 4). The representative sequences of OTUs STR7, PARS26, and PAR37 showed higher levels of identity to those of rice paddy soil clone SP-2-12 (97%) (BAO95989), *M. luteus* (99%) (WP027160273), and *Methylovulum miyakonense* (99%) (WP019865396). The representative sequence of major OTU STR7 (59 out of 250 clones) was also the closest to the known bacterium *M. lenta* (96%) (WP027160273) and *M. denitrificans* strain FJG1 (96%) (AMK77596) (Fig. 4 and 5b).

To elucidate the relationship between methanotrophs and denitrifiers, we compared these two phylogenetic trees based on the *pmoA* and *nirS* genes. The results obtained showed that the three genera, *Methylomonas*, *Methylobacter*, and *Methylovulum*, were frequently detected in both the *pmoA* and *nirS* clone libraries (Fig. 5a and b).

**Fig. 6.**
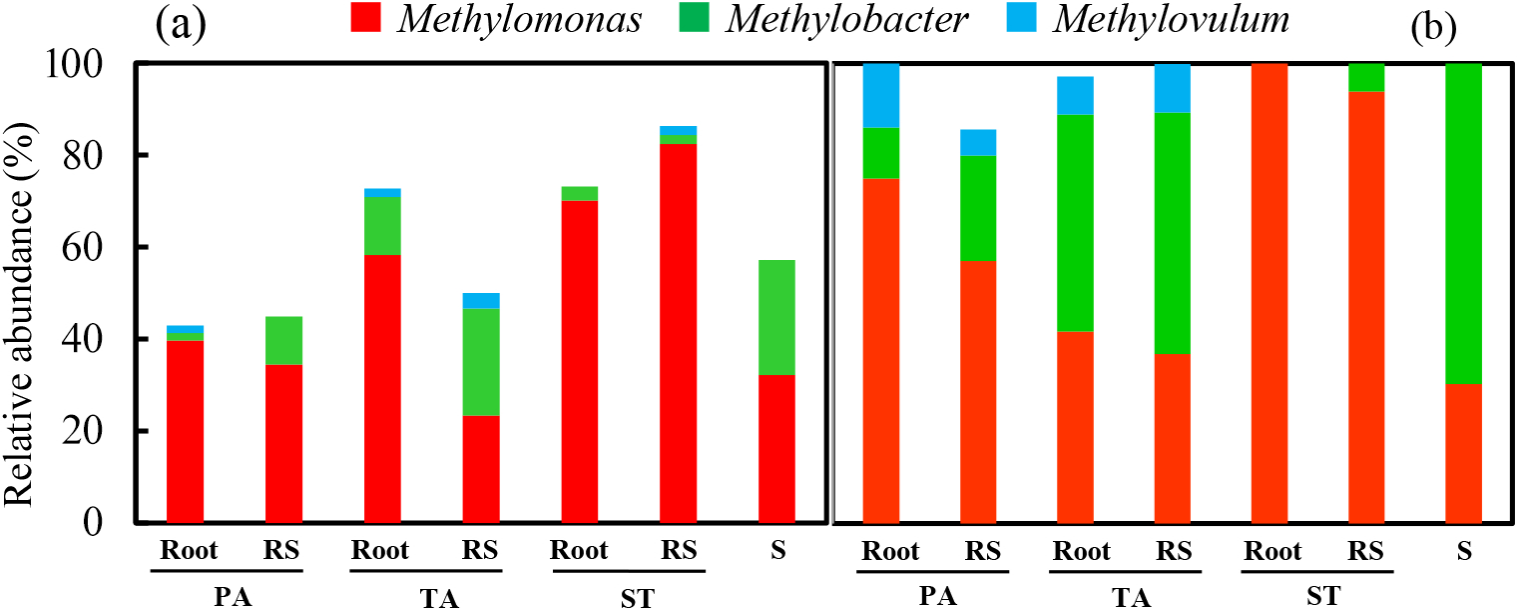
Relative abundance of three common genera of *Methylomonas*, *Methylobacter*, and *Methylovulum* based on *pmoA* (A) and *nirS* (B) gene clone libraries in roots and rhizosphere sediment of three plants and the unvegetated sediment.

We further calculated the relative abundance of the three most common genera in the *pmoA* and *nirS* clone libraries (Fig. 6a and b). *Methylomonas* (34.5–82.4% in the *pmoA* clone library, 57.1–100% in the *nirS* library) was predominant in the PA and ST root zone samples. In the TA root zone and unvegetated sediment samples, *Methylomonas* (23.3–58.2% in the *pmoA* clone library, 30.3–41.7% in the *nirS* library) and *Methylobacter* (12.7–25.0% in the *pmoA* clone library, 47.2–69.7% in the *nirS* library) were both dominant. In ST root zone samples, *Methylobacter* was less frequently detected in both clone libraries. *Methylovulum* (0–3.3% in the *pmoA* clone library and 0–13.9% in the *nirS* library) was detected at smaller numbers than *Methylomonas* and *Methylobacter*. Overall, clones of the *pmoA* and *nirS* genes belonging to *Methylomonas*, *Methylobacter*, and *Methylovulum* of type I methanotrophs were shared by the PA, TA, and ST plants and plant-free sediment. Thus, the relative abundance of *Methylomonas* slightly increased from sediment to rhizosphere sediment and root, whereas that of *Methylobacter* decreased.

The results of PCA clearly showed a tight cluster of community structures between root and rhizosphere sediment for the same plant, whereas distinct separation was observed among the different plant taxa. Differences among plant species were more obvious than those between microhabitats (roots versus the rhizosphere) (Fig. S1a and b).

### Copy numbers of *pmoA*, *nirS*, and *nirK* genes

To estimate the population sizes of methanotrophs and denitrifying bacteria, we performed qPCR assays with the root and rhizosphere sediment of PA, TA, and ST as well as samples of unvegetated sediment. The copy numbers (×10‍^7^‍ ‍g‍^–1^ dry weight) of the *pmoA*, *nirS*, and *nirK* genes in root and sediment samples of the three plant species were calculated, and were significantly higher in rhizosphere sediment than in unvegetated sediment, except for the *pmoA* gene in PA and TA plants (*P*<0.05) (Table 1). Moreover, in comparisons of both *nir* genes in the root zones of the three plant species and in unvegetated sediment, the copy numbers of the *nirS* gene were significantly higher than those of the *nirK* gene (*P*<0.05) (Table 1). Furthermore, Pearson’s linear correlation showed that the copy numbers of the *pmoA* gene positively correlated with those of the *nirS* gene among seven type samples (r=0.741, *P*<0.01, *n*=21). In addition, the copy numbers of both the *pmoA* and *nirS* genes in the roots were higher in PA than in ST and TA plants (Table 1). Combined with the sequencing analysis, these results suggest that the three emergent macrophytes acted selectively on methanotrophs and methanotrophic denitrifiers, particularly *Methylomonas*, *Methylobacter*, and *Methylovulum* of the type I methanotrophs that were enriched in the root zone. Furthermore, the abundance, diversities, and community structures of both groups of microbes varied among plant species.

### Localization of type I methanotrophs in roots of three plant species

To validate our probe, dye, and method, we used type I methanotroph strain *M. koyamae* Fw12E-Y^T^ (NCIMB14606) as a positive control. Clear signals showed that type I methanotrophs in 1a medium were successfully visualized by CARD-FISH (Fig. S2). These signals were not detected from strain *T. thiooxidans*, which was used as a negative control (data not shown).

**Fig. 7.**
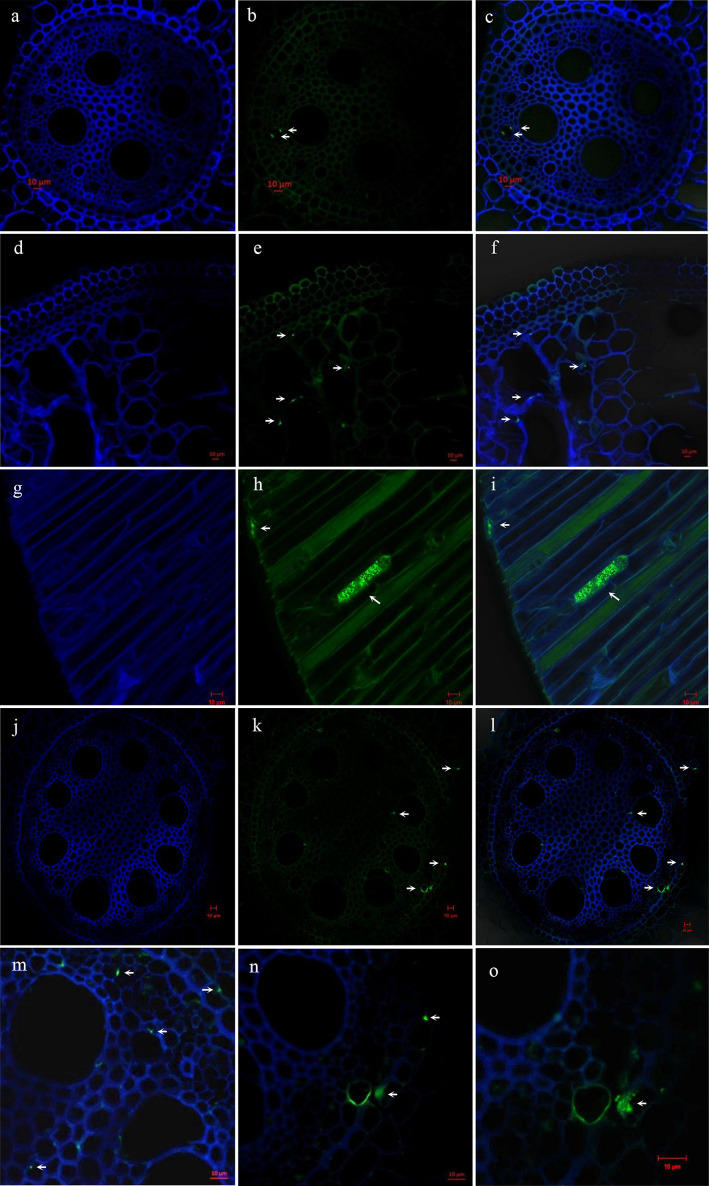
Catalyzed reporter deposition-fluorescence *in situ* hybridization (CARD-FISH) detection of type I methanotrophs in roots of three macrophytes by confocal laser scanning microscopy. a–c, Cross-sections of the stele of a PA root; d–f, cross-sections of the aerenchyma of a PA root; g–i, vertical sections of the ST root; j–o, cross-sections of the stele of a TA root. a–i (×40); j–l (×20); m and n (×63); o (×100); a, d, g, and j: autofluorescence of the cell wall of roots (blue); b, e, h, and k: Alexa Fluor 488 of the Mγ705+Mγ84 probe (green); c, f, i, l, m, n, and o: their overlay. White arrows indicate the detected type I methanotrophs. (PA, *Phragmites australis*; TA, *Typha angustifolia*; ST, *Scirpus triqueter*).

We applied CARD-FISH to the root tissues of the three plant species to identify the localization of type I methanotrophs. Using this method, signals for type I methanotrophs were clearly observed not only in the vascular cylinder (Fig. 7a, b, c, j, k, l, m, n, and o), but also around the aerenchyma (Fig. 7d, e, and f) of PA and TA root tissues. CARD-FISH signals were observed in epithelial cells in the vertical sections of ST root tissues (Fig. 7g, h, and i).

## Discussion

Previous studies demonstrated that the abundance of *pmoA* differed among various genotypes of the rice plant and also that type I methanotrophs were abundant in the roots of rice plants, most likely due to root selection (Wu *et al.*, 2009; Bao *et al.*, 2014b). In addition, type I methanotrophs were found to be more abundant on the surfaces of aquatic plants than type II methanotrophs (Yoshida *et al.*, 2014), which is consistent with the present results (Fig. 2 and Table 1). Moreover, many environmental factors, such as pH and the concentration of NH_4_^+^, influence the community structure of methanotrophs. Molecular evidence has indicated that type I methanotrophs are predominant in the alkaline bodies of water and sediment (Deng *et al.*, 2017). This phenomenon was attributed to the finding that some groups of type I methanotrophs were more likely to reside in saline-alkaline environments (Deng *et al.*, 2017). A previous study suggested that an increase in NH_4_^+^ exerted positive effects on type I members (Mohanty *et al.*, 2006; Yang *et al.*, 2016), whereas type II members were dominant under nitrogen-limited conditions (Graham *et al.*, 1993). WLSH Lake, the location of this study, is a eutrophic alkaline wetland with rising salinity (Table S1), and, thus, may favor the predominance of type I methanotrophs in the root zone of wetland-grown macrophytes (Fig. 2).

*nirS* gene clones were frequently detected as *Methylomonas*, *Methylobacter*, and *Methylovulum* (Fig. 5a and b). These results support previous findings indicating that *M. denitrificans*, *M. methanica*, *M. lenta*, *M. koyamae*, *M. luteus*, and *M. miyakonense* possess functional denitrification genes, including *nirS* (Ren *et al.*, 2000; Hoefman *et al.*, 2014; Padilla *et al.*, 2017). However, *Methyloglobulus*, *Methylococcus*, and *Methylomicrobium* were not detected with the *nirS* primer because these methanotrophs do not possess the *nirS* gene (Padilla *et al.*, 2017) or they were present, but below the level of detection in plant roots.

A distinct separation was found in the community structures of denitrifying methanotrophs among the PA, TA, and ST plants based on PCA (Fig. S1), which is consistent with previous findings showing that plant species affect the microbial community structure in the rhizosphere (Berg and Smalla, 2009). Furthermore, the abundance of *nirS* genes showed that genetic potential was an effective indicator for potential denitrification activity in the roots, and the expression of these genes differed between wetland plant species (Hallin *et al.*, 2015). These differences may be due to differences in root exudates, radial oxygen loss (ROL) rates, and oxygen availability among plant species, which may affect the rhizobacterial community composition and abundance (Calhoun and King, 1997; Wang *et al.*, 2018).

Methane oxidation by aerobic methanotrophs generally occurs in aerobic environments; however, anaerobic and microaerobic conditions are required for denitrification. Rahalkar *et al.* (2009) reported that type I methanotrophs outnumbered type II methanotrophs in anoxic lake sediment by a factor of at least four. Type I methanotrophs were found to be more abundant than type II methanotrophs in the root tissues of rice plants, and this difference was more likely to be related to oxygen concentrations (Wu *et al.*, 2009). Coincidentally, the same finding was reported for the response of pure cultures of type I and II methanotrophs to oxygen concentrations in culture experiments (Graham *et al.*, 1993). In the present study, signals from type I methanotrophs were visualized *in situ* in roots using CARD-FISH, as well as in the epidermal cell walls of *S. triqueter* and around the vascular cylinders of *P. australis* and *T. angustifolia* (Fig. 7). However, Armstrong *et al.* (2010) found that oxygen concentrations at the epidermal/hypodermal cylinder (2 kPa) were lower than those in the root center (>12 kPa) in emergent macrophytes, such as *P. australis*. Kits *et al.* (2015b) reported that methane-dependent *M. denitrificans* strain FJG1 simultaneously performed methane oxidation and denitrification under hypoxia (O_2_ concentration of 1.5%). Therefore, denitrification and CH_4_ oxidation may mainly occur in the epidermis rather than in the vascular cylinder of the roots in type I methanotrophs. To elucidate the underlying mechanisms in more detail, a metagenomic and/or metaproteomic analysis of root-associated microorganisms is needed in order to clarify whether type I methanotrophs play an important role in methane oxidation and denitrification. Furthermore, it will be crucial to characterize the physiological activities of *Methylomonas*, *Methylobacter*, and *Methylovulum* isolates from wetland plants in the future.

In summary, we herein provide the first dataset for the abundance, diversity, and localization of root-associated methanotrophs and denitrifiers in three common emergent macrophytes (*P. australis*, *T. angustifolia*, and *S. triqueter*) in an important eutrophic wetland in northern China. The numbers of *nirS* and *nirK* gene copies were higher in rhizosphere sediment than in unvegetated sediment, suggesting that the presence of macrophytes increased the abundance of both groups of microbes over their levels in plant-free sediments. The abundance and diversities of both groups of microbes varied among plant species, with maximum numbers in *P. australis*, suggesting that macrophyte species had some influence on methanotroph numbers and diversity. The results of the present study emphasized that plant roots were more likely to be enriched with type I methanotrophic denitrifiers, including *Methylomonas*, *Methylobacter*, and *Methylovulum*, which most likely inhabit the epidermal cells, aerenchyma, and vascular bundles of root tissues of three emergent macrophytes due to root selection and environmental selection via excessive nitrogen input and saline-alkaline conditions. The present results suggest that root zone type I methanotrophic denitrifiers are of great importance for simultaneously mediating methane emission and nitrogen removal in vegetated wetlands.

## Supplementary Material

Supplementary Material

## Figures and Tables

**Table 1.** Numbers of *pmoA*, *nirS*, and *nirK* gene copies in roots and rhizosphere sediments of three plants and unvegetated sediment

Genes	Gene abundance in^a^								
	Root (×10^7^)				Rhizosphere sediment (×10^7^)				Unvegetated sediment (×10^7^)
	PA	TA	ST		PA	TA	ST		S
*pmoA*	94.80±37.70	8.38±2.44	40.20±13.10		2.93±2.40	2.01±1.06	8.65±5.97*		3.35±0.68
*nirS*	14.60±5.33^##^	6.51±1.79^#^	17.20±6.88^##^		1.20±0.57*^#^	1.27±0.10*^##^	1.90±0.40**^##^		0.78±0.08^##^
*nirK*	3.60±1.12	2.87±0.45	2.02±0.52		0.20±0.02**	0.14±0.08**	0.23±0.10**		0.05±0.02

^a^ Values are means±standard errors of gene abundance levels (in copies g^–1^ dry weight) obtained from three independent DNA extracts from the roots and rhizosphere sediments of three plants and unvegetated sediment. Significant differences between rhizosphere sediments and unvegetated sediment are indicated by an asterisk (* *P*<0.05; ** *P*<0.01); significant differences between *nirS* and *nirK* genes in the same sample are indicated by a pound sign (^#^ *P*<0.05; ^##^ *P*<0.01). (PA, *Phragmites australis*; TA, *Typha angustifolia*; ST, *Scirpus triqueter*; R, root; RS, rhizosphere sediment; S, unvegetated sediment).
