# Supplementary material for: Enrichment of Type I Methanotrophs with nirS Genes of Three Emergent Macrophytes in a Eutrophic Wetland in China
Source: Microbes Environ. 2020 Jan 23;35(1):ME19098. doi: 10.1264/jsme2.ME19098 (PMC7104278; doi:10.1264/jsme2.ME19098)
Supplement: Supplementary file 1 — Supplementary Material [file 35_19098_s1.pdf]

Enrichment of type I methanotrophs with *nirS* genes of three emergent macrophytes in a eutrophic wetland in China

Supplementary files

Jumei Liu[1,4], Zhihua Bao[2,3*], Weiwei Cao[2,5], Jingjing Han[2], Jun Zhao[2], Zhenzhong Kang[2], Lixin Wang[2], Ji Zhao[2,3*]

1 College of Life Sciences, Inner Mongolia University, Hohhot 010021, China
2 Ministry of Education Key Laboratory of Ecology and Resource Use of the Mongolian Plateau & Inner Mongolia Key Laboratory of Grassland Ecology, School of Ecology and Environment, Inner Mongolia University, Hohhot, 010021, China
3 Inner Mongolia Key Laboratory of Environmental Pollution Control & Waste Resource Reuse, Inner Mongolia University, Hohhot 010021, China
4 College of Chemistry and Environmental Engineering, Chongqing Key Laboratory of Environmental Materials & Remediation Technologies, Chongqing University of Arts and Sciences, Chongqing, 402160, China.

*Corresponding author: Zhihua Bao, Ji Zhao; School of Ecology and Environment, Inner Mongolia University, 235 West University Street, Hohhot 010021, China. Phone: +86-471-499-1469. E-mail: zhihua_bao@imu.edu.cn; ndzj@imu.edu.cn.

Table S1. Environmental data for sampling site (latitude, 40º52′35.00″ N; longitude, 108º51′15.00″ E)

Environmental data		Samples	
		Sediment[a]	Water[b]
Chemical properties			
	EC(ds m^{-1})	1.35±0.22	6.25±0.07
	pH	9.00±0.06	9.30±0.03
	TOC	18.35±1.45	27.62±1.45
	TN	2.60±0.43	1.87±0.15
	TP	0.62±0.05	0.03±0.00
	NH_4^+-N	24.45±1.63	0.30±0.01
	NO_3^--N	1.28±0.14	0.19±0.02
Soil texture (Sandy loam)			
	Clay (%)	0.96±0.17	—
	Silt (%)	17.65±1.00	—
	Sand (%)	81.40±1.16	—

[a]: g kg^{-1} for TOC, TN and TP; mg kg^{-1} for NH_4^+-N and NO_3^--N

[b]: mg L^{-1} for TOC, TN, TP , NH_4^+-N and NO_3^--N

EC, electrical conductivity; TOC, total organic carbon; TN, total nitrogen; TP, total phosphorus; NH_4^+-N, ammonium nitrogen; NO_3^--N, nitrate nitrogen.

Table S2. Statistical analysis of *pmoA* and *nirS* genes clone libraries derived from the root zone of three plants and unvegetated sediment

Parameter	*pmoA* gene							*nirS* gene						
	Root			Rhizosphere sediment			Unvegetated sediment	Root			Rhizosphere sediment			Unvegetated sediment
	PA	TA	ST	PA	TA	ST	S	PA	TA	ST	PA	TA	ST	S
No. of clones	63	55	67	29	30	51	56	36	36	39	35	38	33	33
Coverage %	82.5	89.1	92.5	72.4	80	86.3	87.5	77.78	86.11	100	91.43	86.84	93.94	96.97
No. of OTUs	16	14	11	11	12	9	15	14	14	3	15	13	5	8
Diversity indexes														
Chao Ⅰ	43.5	19	14.3	25	17	30	20.3	28	16.5	3	15.38	15.5	5.5	8
ACE	87.73	18.99	23.76	89.02	19.14	74.05	35.14	40.04	17.34	3.00	16.41	18.41	12.58	8.56
Shannon index	1.87	1.95	1.33	1.84	2.22	0.88	2.22	2.28	2.45	0.99	2.59	2.27	1.06	1.87
Simpson index (1/D)	4.00	4.17	2.27	5.00	10.00	1.54	7.69	9.09	14.29	2.63	16.67	9.09	2.44	6.25

Note PA, *Phragmites australis*; TA, *Typha angustifolia*; ST, *Scirpus triqueter*; R, root; RS, rhizosphere sediment; S, unvegetated sediment.

Table S3. PCR amplification primers and reaction conditions

Target gene	Primer Name	Primer sequence (5'-3')	Length of amplicon (bp)	Quantitative PCR	References
pmoA	A189F	GGN GAC TGG GAC TTC TGG	508	95℃, 30 s; 35×(95℃, 30 s; 53℃, 45 s; 72℃, 45 s with plate read); Melt curve 65.0℃ to 95.0℃, increment 0.5℃, 0:05+ plate read	Costello and Lidstrom (1999)
	mb661R	CCG GMG CAA CGT CYT TAC C			
nirS	nirSC2F	TGG AGA ACG CCG GNC ARG TNT GG	410-420	98℃, 30 s; 39×(98℃, 10 s; 55℃, 10 s; 68℃, 30 s with plate read); Melt curve 65.0℃ to 95.0℃, increment 0.5℃, 0:05+ plate read	Wei et al. (2015)
	nirSC2R	GAT GAT GTC CAC GGC NAC RTA NGG			
nirK	F1aCu	ATC ATG GTS CTG CCG CG	473	98℃, 30 s; 40×(98℃, 10 s; 58℃, 10 s; 68℃, 30 s with plate read); Melt curve 65.0℃ to 95.0℃, increment 0.5℃, 0:05+ plate read	Hallin and Lindgren (1999)
	R3Cu	GCC TCG ATC AGR TTG TGG TT			

Costello, A.M., M. Lidstrom. 1999. Molecular characterization of functional and phylogenetic genes from natural populations of methanotrophs in lake sediments. Appl. Environ. Microbiol. 65(11):5066-5074.

Wei, W., K. Isobe, T. Nishizawa, L. Zhu, Y. Shiratori, N. Ohte, K. Koba, S. Otsuka, and K. Senoo. 2015. Higher diversity and abundance of denitrifying microorganisms in environments than considered previously. ISME J. 9(9):1954-1965.

Hallin, S., P.E. Lindgren. 1999. PCR detection of genes encoding nitrite reductase in denitrifying bacteria. Appl. Environ. Microbiol. 65(4):1652-1657.

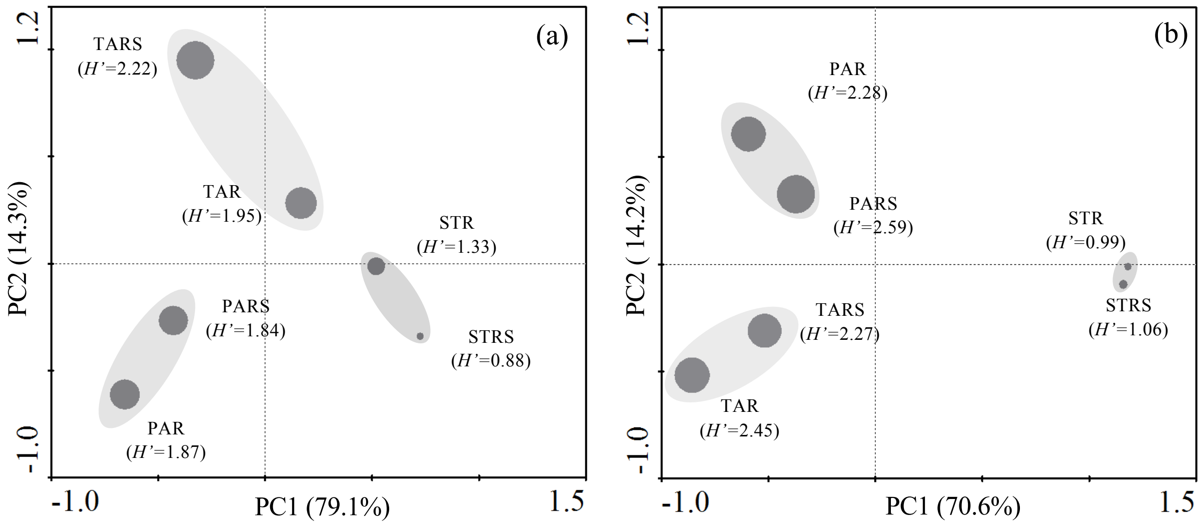

Fig. S1. Principal-component analysis (PCA) based on *pmoA* (a) and *nirS* (b) gene clone library sequences for both communities of methanotrophs and methanotrophic denitrifiers in roots and rhizosphere soils of three plants (PA, *Phragmites australis*; TA, *Typha angustifolia*; ST, *Scirpus triqueter*; R, root; RS, rhizosphere sediment). The size of each gray circle indicates the Shannon index (*H'*) for that data point.

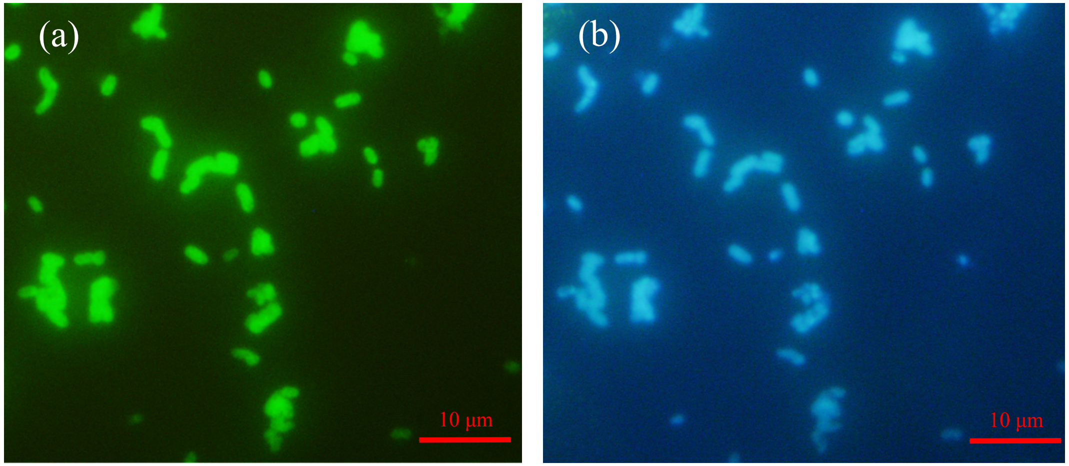

Fig. S2. Catalyzed reporter deposition fluorescence in situ hybridization (CARD-FISH) detection of the type strain *Methylomonas koyamae* Fw-12^{T} (type I methanotrophs). Micrographs of in situ hybridization with probe Mγ705+Mγ84 (a) and 4’, 6-diamidino-2-phenylindole staining (b)
